# Supplementary material for: NLRP12 suppresses hepatocellular carcinoma via downregulation of cJun N-terminal kinase activation in the hepatocyte
Source: eLife. 2019 Apr 16;8:e40396. doi: 10.7554/eLife.40396 (PMC6483596; doi:10.7554/eLife.40396)
Supplement: Supplementary file 1. — All primers used in this study to measure the expression of mouse genes (Supplemental Table 1) and of human genes (Supplemental Table 2) by real-time qPCR analysis. [file elife-40396-supp1.docx]

**Supplemental Table 1.** Primer sequences for real-time qPCR analyses of mouse genes.

| **Genes Name** | **Genes Primer sequences** |
| --- | --- |
| GAPDH_F | TGGCAAAGTGGAGATTGTTGCC |
| GAPDH_R | AAGATGGTGATGGGCTTCCCG |
| Il1β_F | GCCTCGTGCTGTCGGACCCATA |
| Il1β_R | TGCAGGGTGGGTGTGCCGTCTT |
| Il6_F | CAAGAAAGACAAAGCCAGAGTC |
| Il6_R | GAAATTGGGGTAGGAAGGAC |
| Ccl2_F | TTGCCGGCTGGAGCATCCACGT |
| Ccl2_R | AGTAGCAGCAGGTGAGTGGGGCG |
| Cxcl1 _F | TGAGCTGCGCTGTCAGTGCCT |
| Cxcl1_R | AGAAGCCAGCGTTCACCAGA |
| Cxcl2_F | CAAGAACATCCAGAGCTTGAGTGT |
| Cxcl2_R | GCCCTTGAGAGTGGCTATGACTT |
| Cox-2_F | CCAGAGCAGAGAGATGAA |
| Cox-2_R | GGTACAGTTCCATGACATC |
| Ki67_F | AGAAGTCCAGGTCTACAG |
| Ki67_R | TCGTTGCTATTGCTAAGG |
| Nlrp12_F | CCT CTT TGA GCC AGA CGA AG |
| Nlrp12_R | GCC CAG TCC AAC ATC ACT TT |
| Emr1_F | CCTGGACGAATCCTGTGAAG |
| Emr1_R | GGTGGGACCACAGAGAGTTG |
| Cdkn1a_F | GCAGATCCACAGCGATATCC |
| Cdkn1a_R | CAACTGCTCACTGTCCACGG |
| Il4_F | AGATGGATGTGCCAAACGTCCTCA |
| Il4_R | AATATGCGAAGCACCTTGGAAGCC |
| Il17_ | TGAGCTTCCCAGATCACAGA |
| Il17_R | TCCAGAAGGCCCTCAGACTA |
| Afp_F | CTGGCGATGGGTGTTTAGAA |
| Afp_R | GCCTGAGAGTCCATACTTGTTAG |
| Ccnd1_F | TGCCATCCATGCGGAAA |
| Ccnd1d1 R | AGCGGGAAGAACTCCTCTTC |
| Myc_F | CCTTTGGGCGTTGGAAACC |
| Myc_R | TCCTCGTCGCAGATGAAATAGG |
| Ifng_F | GAAAGACAATCAGGCCATCA |
| Ifng_R | TTGCTGTTGCTGAAGAAGGT |
| Ccnb1_F | AAAGGGAAGCAAAAACGCTAGG |
| Ccnbb1_R | TGTTCAAGTTCAGGTTCAGGCTC |
| Survivin_F | TCATCCACTGCCCTACCGAGAAC |
| Survivin_R | TCTATCGGGTTGTCATCGGGTTC |

**Supplemental Table 2.** Primer sequences for real-time qPCR analyses of human genes.

| **Genes Name** | **Genes Primer sequences** |
| --- | --- |
| GAPDH_F | TCTGGTAAAGTGGATATTGTTG |
| GAPDH_R | GATGGTGATGGGATTTCC |
| IL-6_F | GTAGCCGCCCCACACAGA |
| IL-6_R | CATGTCTCCTTTCTCAGGGCTG |
| CCL2_F | AGGTGACTGGGGCATTGAT |
| CCL2_R | GCCTCCAGCATGAAAGTCTC |
| CXCL1 _F | AACCGAAGTCATAGCCACAC |
| CXCL1_R | CCTCCCTTCTGGTCAGTT |
| CXCL2_F | CGCCCAAACCGAAGTCAT |
| CXCL2_R | GATTTGCCATTTTTCAGCATCTTT |
| COX-2_F | CCCATGTCAAAACCGAGGTG |
| COX-2_R | CCGGTGTTGAGCAGTTTTCTC |
| Ki67_F | GAGGTGTGCAGAAAATCCAAA |
| Ki67_R | CTGTCCCTATGACTTCTGGTTGT |
| NLRP12_F | AAATGCACTGGAGGATTTGG |
| NLRP12_R | CAGGCTCTGGTTCACACTGA |
